# Continuous-Variable Quantum Secret Sharing Through Microwave-Enabled Turbulent Channels with Measurement-Device-Independent Scheme

**DOI:** 10.3390/e28050540

**Published:** 2026-05-10

**Authors:** Weihan Zhang, Zhangtao Liang, Yun Mao, Hang Zhang, Ying Guo

**Affiliations:** 1 School of Computer Science, Beijing University of Posts and Telecommunications, Beijing 100876, China; 2 School of Automation, Central South University, Changsha 410083, China; 3 Provincial Key Laboratory of Informational Service for Rural Area of Southwestern Hunan, College of Information Science and Engineering, Shaoyang University, Shaoyang 422000, China

**Keywords:** continuous-variable quantum secret sharing, measurement-device-independent, turbulent channels, microwave, Kolmogorov turbulence, Shamir threshold scheme

## Abstract

Quantum secret sharing (QSS) has been previously demonstrated with conceivability in optical-fiber channels. However, extending this framework to the microwave frequency band presents challenges in achieving secure quantum communications over turbulent channels, as intricate turbulence can induce amplitude and phase jitter in quantum signals, leading to decoherence or even interruptions in the communication link. In this work, we propose a microwave-enabled continuous-variable quantum secret sharing (CVQSS) scheme operating over turbulent free-space channels. The protocol explicitly addresses the extreme sensitivity of microwave quantum states to environmental turbulence, which manifests as severe amplitude and phase fluctuations. It incorporates the Shamir threshold scheme to facilitate multi-user secret sharing. We suggest a flexible approach to solving problems of adaptive phase compensation and multi-aperture reception techniques when characterizing an equivalent noise channel based on the Kolmogorov turbulence model. The proposed measurement-device-independent (MDI) architecture renders the protocol immune to all detector-side attacks, provided that the state preparation at the users’ side is trusted. Numerical simulations ascertain the performance of the microwave continuous-variable measurement-device-independent quantum secret sharing (CV-MDI-QSS) system and demonstrate the feasibility of practical deployment in complicated turbulent channels. This approach offers a turbulence-resistant solution for dynamic quantum networks through harsh free-space channels implemented in microwave-propagated environments.

## 1. Introduction

Quantum secret sharing (QSS) extends the concept of secret sharing from classical cryptography into the quantum realm [1]. It aims to securely distribute a quantum or classical secret among multiple participants while ensuring that only an authorized subset (e.g., reaching a specific threshold) can collaboratively recover the secret [2]. As a fundamental building block for constructing future secure quantum networks, involving the distributed quantum computing, quantum blockchains, and fault-tolerant quantum cryptographic systems, the implementation significance of practical QSS is undeniable [3,4]. Previous QSS schemes have predominantly relied on discrete-variable (DV) encoding [5], such as the polarization state of single photons [6,7]. However, these approaches are typically highly sensitive to channel loss and noise, and their implementation often requires efficient single-photon sources and detectors, posing challenges for large-scale practical applications [8,9].

In contrast, continuous-variable quantum secret sharing (CVQSS) utilizes orthogonal components of the light field, such as amplitude and phase, to encode information [10,11]. Its core advantage lies in compatibility with mainstream classical optical communication equipment, enabling high-rate key distribution in practice [12]. Traditional CVQSS protocols require all participants to sequentially transmit quantum states through a shared quantum channel [13]. However, this architecture suffers from inherent flaws. For example, it assumes all measurement devices in the network are fully trusted, which is a requirement often difficult to meet in practice due to potential backdoor implantation or targeted attacks [14]. Additionally, excessive noise introduced into the intricate channel accumulates sequentially among users, severely limiting network scalability and performance [15].

Intriguingly, to address the vulnerabilities in measurement devices, measurement-device-independent (MDI) protocols were proposed and rapidly gained prominence in quantum communications [16]. The idea of MDI protocols is to delegate all complex measurement operations to an untrusted third party (Charlie), while user terminals (Alice, Bob, etc.) only need to prepare and transmit quantum states [17,18]. This architecture concentrates all detector vulnerabilities onto the third party, ensuring protocol security as long as the user’s source remains trustworthy [19,20]. As a consequence, integrating the MDI framework with CVQSS to form CV-MDI-QSS enables the construction of inherently detector-resistant multi-user secure networks, representing a promising development direction in this work.

Although CV-MDI-QSS has demonstrated potential in fiber-optic channels, its application to free-space channels (such as satellite relays and drone swarm networking) faces the severe challenge of atmospheric turbulence [21,22]. Random fluctuations in the refractive index caused by turbulence lead to beam spreading, drift, intensity scintillation (scintillation index SI), and phase distortion [23,24]. These effects result in feasible quantum state decoherence, manifesting as severe fluctuations in channel transmission efficiency and a significant increase in excess noise, thereby limiting the secret key rate and transmission distance of secret sharing [25,26,27]. Specifically, implementing CV-MDI-QSS in the microwave band (1–100 GHz) offers several advantages, such as strong penetration, broad beam coverage, and ease of integration with classical wireless infrastructure [28,29]. However, the low energy of microwave photons makes them highly susceptible to being drowned out by environmental thermal noise, imposing extremely stringent demands on quantum state preparation, transmission, and detection.

Currently, implementing CV-MDI-QSS into microwave-propagated turbulent channels has rarely been touched upon. Most existing work either relies on an ideal channel assumption or fails to systematically address the dynamic degradation caused by turbulence [30]. To fill this gap, this paper proposes and validates the feasible microwave-band CV-MDI-QSS scheme tailored for the turbulent channels [31]. Notably, we present a microwave CV-MDI-QSS framework that seamlessly integrates Shamir’s secret sharing scheme into the quantum key distribution (QKD) process. Simultaneously, we establish an equivalent noise channel based on the Kolmogorov turbulence model, providing a theoretical tool for analyzing turbulence’s impact on the performance of the CV-MDI-QSS. Meanwhile, we design and validate a combined anti-turbulence strategy for pre-compensated coding–multi-aperture reception–dynamic post-selection, resulting in enhanced system robustness.

For clarity, we explicitly separate the protocol definition, channel modeling, noise and security evaluation, and numerical performance validation into different sections. The protocol description is independent of any specific channel assumptions, while the effects of turbulence and noise are incorporated only at the channel and security analysis levels. Numerical simulations are finally used to validate the analytical results under realistic conditions.

To contextualize our design, recent advances in CVQSS have demonstrated significant progress, including local local oscillator (LLO) configurations [32] and advanced modulation frameworks [33,34]. While discrete-variable microwave quantum communication has seen experimental breakthroughs using superconducting qubits, continuous-variable implementations remain scarce. Implementing our theoretical framework relies heavily on the feasibility of existing microwave quantum technology. Although microwave quantum states are acutely sensitive to thermal noise, modern superconducting circuits—specifically Josephson Parametric Amplifiers (JPAs) operating at the quantum noise limit and sophisticated High-Electron-Mobility Transistor (HEMT) amplifiers—provide a realistic hardware foundation for our protocol.

## 2. Microwave-Enabled CV-MDI-QSS

This section suggests an approach to achieving CVQSS in the microwave frequency band at millikelvin temperatures. While leveraging the inherent robustness of microwave signals against environmental interference, we demonstrate superior stability of superconducting quantum state preparation compared to protocols based on short-wavelength optical quantum signals. This system adopts the MDI architecture, delegating quantum state measurement tasks to untrusted third-party nodes [35]. Accordingly, it eliminates all detector-side channel attacks, enhancing the practical security of the CVQSS scheme [36].

### 2.1. CV-MDI-QSS Model

To begin with, for microwave source preparation, we generate Gaussian-modulated microwave coherent states using superconducting circuits, which can suppress spatial pattern perturbations caused by turbulence through multi-aperture reception and maximum ratio combining algorithms. During data processing, the generated shared key will be partitioned into multiple subkeys via the Shamir thresholding scheme and then distributed to participants via microwave links. This design not only addresses signal loss during free-space microwave transmission but also achieves stable multi-user secret sharing in short-range scenarios through a quantum-classical hybrid architecture.

**Figure 1:**
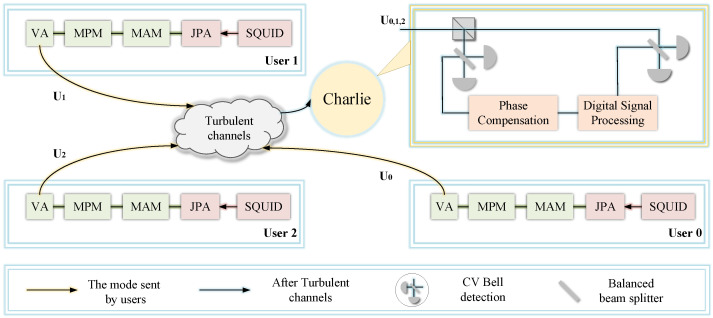
Schematic of microwave CV-MDI-QSS based on turbulent channels. Three remote users (User 1, User 2 and User 0) are connected to Charlie in an ordered manner through a untrusted quantum channel. Each user locally generates 10 GHz microwave coherent states through a superconducting quantum interference device (SQUID), a Josephson Parameter Amplifier (JPA), a microwave amplitude modulator (MAM), a microwave phase modulator (MPM), a variable attenuator (VA), and sends them to Charlie. The FPGA controller synchronizes the clocks for User 1, User 2, User 0 and Charlie, performs turbulence monitoring, and measures $C_{n}^{2}$ in real time.

Without loss of generality, we consider three legal participants, called User 1, User 2, and User 0, in the CV-MDI-QSS scheme implemented through microwave-propagated turbulent channels. A schematic of CV-MDI-QSS is shown in Figure 1. User 1 generates microwave pulses using their own superconducting quantum interference device (SQUID) and Josephson Parameter Amplifier (JPA) [37], which are generated through a microwave amplitude modulator (MAM), a microwave phase modulator (MPM), and a variable attenuator (VA) to produce the microwave coherent states. The output quantum state of User 1 can be represented as $|x_{1}+ip_{1}\rangle$, where the quadratures $x_{1}$ and $ip_{1}$ are independently drawn from zero-mean Gaussian distributions with variance *V*. The prepared quantum signals are transmitted through the turbulent channel to the relay.

Translating these quantum states from a controlled cryogenic environment to a turbulent free-space channel presents distinct engineering challenges. The SQUIDs and JPAs must be strictly maintained at millikelvin temperatures within a dilution refrigerator to prevent thermal decoherence. To bridge this with the ambient free-space environment, the quantum signals are routed through a series of thermalized, low-loss microwave windows and heavily attenuated coaxial lines. This careful cryogenic integration ensures that the initial state preparation remains strictly isolated from ambient thermal leaks.

Similarly, User 2 prepares independent Gaussian-modulated microwave coherent states $|x_{2}+ip_{2}\rangle$. In addition to the quantum signals, User 2 generates reference signals that are used for relative phase estimation between the users. The quantum and reference signals are multiplexed and transmitted through the same turbulent channel, ensuring that they experience identical channel fluctuations.

User 0 (the dealer) prepares microwave coherent states using the same Gaussian modulation scheme. The dealer’s signals are transmitted to Charlie, where they interfere with the incoming signals from the participants as part of a continuous-variable Bell measurement. At the relay, the relative phases between different users are estimated using the reference signals, enabling phase compensation for fast channel-induced phase drifts.

After phase compensation, Charlie performs a continuous-variable Bell measurement and publicly announces the measurement outcomes through an authenticated classical channel. Based on the announced results and their locally stored modulation data, the users obtain correlated classical variables. These data are subsequently processed via classical post-processing and a threshold quantum secret sharing protocol to establish shared secret keys.

For data post-processing, we use an FPGA-controlled pre-compensation module for real-time phase pre-distortion loading based on the pilot signal, with a delay of less than 10 μs. Additionally, we perform orthogonal component joint measurements of the generated microwave coherent states using a superconducting microwave mixer and utilize a superconducting antenna array in conjunction with maximum ratio combining (MRC) to suppress spatial mode perturbations. Analogously, by heating the sheet material with a fan, convective vortices can be generated to simulate atmospheric turbulence effects denoted by the refractive index $C_{n}^{2}$. Moreover, we have time-varying amplitude undulations and phase noise based on the Kolmogorov spectral model with the scintillation index SI and transmittance η. In what follows, we will show the effect of these parameters on the performance of the proposed scheme.

### 2.2. Threshold-Based QSS Scheme

To ensure clarity, the execution of the microwave CV-MDI-QSS protocol can be summarized in the following step-by-step procedure: All participants, including the dealer (User 0), prepare independent Gaussian-modulated microwave coherent states. Users transmit their respective modes through the turbulent free-space channel to Charlie. The dealer does not participate in the measurement process. Charlie, an untrusted third party, performs continuous-variable Bell measurements on the received multiplexed signals and publicly announces the classical outcomes. Based on Charlie’s broadcast, the users and dealer perform parameter estimation, reverse reconciliation, and privacy amplification to extract correlated key rates.

All users prepare microwave coherent states with variance $V_{A}$ and send them to Charlie. At Charlie’s location, the dealer input pattern is split into n patterns through the rational deployment of beam splitters (BS). Each user pattern is mixed with one of the dealer patterns, and Charlie performs CV Bell detection on these mixed patterns. Assuming the *i*th user mode ($User_{i}$) is mixed with the dealer mode through *k* BSs, the measurement result of these modes can be expressed as:

(1) $$\begin{array}{c} \left(\hat{Q},\hat{P}\right)=\left({\hat{Q}}_{U_{i}}-{\left(\frac{1}{\sqrt{2}}\right)}^{k}{\hat{Q}}_{D},{\hat{P}}_{U_{i}}+{\left(\frac{1}{\sqrt{2}}\right)}^{k}{\hat{P}}_{D}\right). \end{array}$$

The subsequent measurement result is then broadcast.

Based on Charlie’s measurement result, the dealer and users reveal partial data to estimate the channel transmission rate and excess noise, where the revealed data is discarded.The dealer subtracts its own data from the broadcast measurement results to estimate the user data, thereby obtaining the data,

(2) $$\begin{array}{c} \left({\hat{Q}}_{D_{i}}^{\prime },{\hat{P}}_{D_{i}}^{\prime }\right)=\left(\hat{Q}+{\left(\frac{1}{\sqrt{2}}\right)}^{k}\sqrt{T_{dc}}{\hat{Q}}_{D}^{s},\hat{P}-{\left(\frac{1}{\sqrt{2}}\right)}^{k}\sqrt{T_{dc}}{\hat{P}}_{D}^{S}\right). \end{array}$$

where $T_{dc}$ is the estimated channel transmission rate between the dealer and Charlie. This establishes a point-to-point measurement-device-independent continuous-variable quantum key distribution (MDI-CVQKD) link. Consequently, established MDI-CVQKD security analysis techniques can be applied to estimate the lower bound of the key rate $R_{i}$ between the dealer and the user. After repeating the above steps three times, three estimated key rates $\left\{R_{0},R_{1},R_{2}\right\}$ are obtained. To ensure system security, the minimum key rate should be selected as the final key rate for CV-MDI-QSS, denoted as $R=\min\{R_{0},R_{1},R_{2}\}$.

If the *R* value is positive, dealers and users can generate keys based on their respective undisclosed raw data through mature data coordination and privacy-enhancing technologies. Rather than a trivial (3,3) collaboration, our system employs a generalized (t,n) Shamir threshold scheme. The dealer constructs a random polynomial f(x) of degree t−1, where the constant term f(0)=S represents the underlying secret key. The dealer then distributes the shares f(i) to the *n* users through the securely established MDI-CVQKD links. Consequently, any authorized subset of *t* or more users can collaboratively reconstruct the polynomial via Lagrange interpolation to recover *S*, while any group of t−1 or fewer users gains zero information regarding the secret.

The Shamir scheme inherently possesses information-theoretic security. Any combination of fewer than *k* shares cannot reveal any information about the secret key *s*. Even if a participant’s share is compromised or a node’s key storage is breached, the overall key remains secure as long as the number of compromised shares does not reach the threshold *k*. All shares are generated and verified locally by users, eliminating the risk of a single administrator holding the complete key. This scheme embeds the Shamir threshold mechanism into the key generation process of CV-MDI-QSS, enabling the entire system to achieve secure and robust secret sharing among multiple users while inheriting the MDI architecture’s resistance to probe attacks.

## 3. Channel Loss and Noise Analysis

Although continuous-variable quantum secret sharing has been extensively analysed in optical-fibre channels, its extension to microwave frequencies propagating through turbulent free-space links introduces a unique interplay of deterministic geometric loss, spectrally selective gaseous absorption, and stochastic refractive-index fluctuations that collectively govern the attainable secret key rate. In the present microwave CV-MDI-QSS architecture, wherein Gaussian-modulated coherent states are prepared with superconducting circuitry and detected at an untrusted relay after traversal of Kolmogorov-type turbulence, we derive the composite channel transmissivity and associated excess noise from first principles. The microwave regime—owing to its comparatively long wavelength—exhibits markedly reduced sensitivity to amplitude scintillation while remaining susceptible to thermal photon loading, a limitation that is nevertheless ameliorated by high-gain antennas, multi-aperture reception, and real-time phase compensation. We begin with the decomposition of the total transmissivity η, which multiplies four independent contributions:

(3) $$\begin{array}{c} \eta =\eta_{\mathrm{eff}}\cdot \eta_{\mathrm{geo}}\cdot \eta_{\mathrm{atm}}\cdot \eta_{\mathrm{TB}}. \end{array}$$

Here $\eta_{\mathrm{eff}}$ denotes the receiver efficiency (the fraction of incident power actually coupled into the detection chain), $\eta_{\mathrm{geo}}$ is the geometric spreading transmissivity arising from free-space diffraction, $\eta_{\mathrm{atm}}$ is the deterministic atmospheric gaseous absorption transmissivity, and $\eta_{\mathrm{TB}}$ is the stochastic turbulence-induced transmissivity. The geometric contribution follows directly from the Friis transmission equation, which equates the received power $P_{r}$ to the transmitted power $P_{t}$ modified by the transmit antenna gain $G_{t}$, the receive antenna gain $G_{r}$, the wavelength λ of the carrier, and the propagation distance *d*:

(4) $$\begin{array}{c} P_{r}=P_{t}\;G_{t}\;G_{r}{\left(\frac{\lambda }{4\pi d}\right)}^{2}. \end{array}$$

The corresponding transmissivity is therefore

(5) $$\begin{array}{c} \eta_{\mathrm{geo}}={\left(\frac{\lambda }{4\pi d}\right)}^{2}, \end{array}$$

or, in the logarithmic form commonly used for link-budget calculations,

(6) $$\begin{array}{c} L_{p}=-10\log_{10}\eta_{\mathrm{geo}}, \end{array}$$

where $L_{p}$ is the free-space path loss expressed in decibels. The additional loss term $L_{a}$ (accounting for any non-diffraction mechanisms) appears in the power balance equation

(7) $$\begin{array}{c} P_{r}=P_{t}+G_{t}+G_{r}-L_{p}-L_{a}, \end{array}$$

so that the full geometric-plus-additional transmissivity is $\eta_{\mathrm{geo}}\cdot 10^{-L_{a}/10}$. Superimposed upon geometric spreading is atmospheric gaseous absorption, quantified by the specific attenuation $\gamma_{\mathrm{total}}$ (in dB per unit distance) according to the ITU-R model:

(8) $$\begin{array}{c} \gamma_{\mathrm{total}}=\gamma_{o}+\gamma_{w}, \end{array}$$

where $\gamma_{o}$ is the oxygen contribution and $\gamma_{w}$ is the water vapor contribution. The corresponding transmissivity is

(9) $$\begin{array}{c} \eta_{\mathrm{atm}}=10^{-\gamma_{\mathrm{total}}\;d/10}, \end{array}$$

where *d* is again the propagation distance. Both $\gamma_{o}$ and $\gamma_{w}$ are functions of the carrier frequency, pressure, temperature, and water vapor density, but their explicit forms are not required for the present symbolic treatment. The stochastic component arises from Kolmogorov turbulence. Starting from the paraxial wave equation under the Markov approximation, the complex field amplitude ψ=exp(χ+iϕ) experiences log-amplitude fluctuations χ whose variance satisfies

(10) $$\begin{array}{c} \sigma_{\chi }^{2}=0.307\;C_{n}^{2}\;k^{7/6}\;d^{11/6}, \end{array}$$

where $C_{n}^{2}$ is the refractive-index structure constant, k=2π/λ is the wave number, and *d* is the propagation distance. The intensity scintillation index is then

(11) $$\begin{array}{c} \mathrm{SI}=\exp\left(4\sigma_{\chi }^{2}\right)-1\approx 1.23\;C_{n}^{2}\;k^{7/6}\;d^{11/6}\equiv \sigma_{R}^{2}, \end{array}$$

the classical Rytov variance. In the weak-turbulence regime ($\sigma_{R}^{2}\ll 1$), the turbulence-induced transmissivity $\eta_{\mathrm{TB}}$ obeys a log-normal distribution:

(12) $$\begin{array}{c} \eta_{\mathrm{TB}}=\exp\left(-\frac{\sigma_{\ln\eta }^{2}}{2}+\sigma_{\ln\eta }\;X\right),\;X∼N\left(0,1\right), \end{array}$$

where $\sigma_{\ln\eta }=\sqrt{\ln(1+\mathrm{SI})}$. Ensemble averaging yields the effective mean transmissivity $\tau_{E}=\langle \eta_{\mathrm{atm}}\;\eta_{\mathrm{TB}}\rangle$, which enters all subsequent security calculations. Turning to noise, the dominant contribution is thermal occupation of the vacuum mode. From Planck’s law, the mean photon number per mode is

(13) $$\begin{array}{c} n_{\mathrm{th}}=\frac{1}{\exp(hf/k_{B}T)-1}, \end{array}$$

where *h* is Planck’s constant, *f* is the carrier frequency, $k_{B}$ is Boltzmann’s constant, and *T* is the absolute temperature.

At T=300 K and f=10 GHz, $n_{\mathrm{th}}$ exceeds 600 photons per mode, threatening to completely obscure the quantum signal. To counter this extreme thermal loading, our protocol mandates the use of cryogenic receiver front-ends, high-directivity antenna arrays ($G_{r}\gg 1$), and phase-sensitive heterodyne detection, which effectively restricts the thermal acceptance bandwidth and drastically suppresses background photons entering the signal mode prior to covariance evaluation. After propagation through a lossy channel, the average added noise photons become

(14) $$\begin{array}{c} \bar{n}=\frac{1-\tau_{E}}{2}\;n_{\mathrm{th}}. \end{array}$$

The entangling-cloner attack (modelled by an EPR pair of variance *N*) injects additional excess noise

(15) $$\begin{array}{c} \epsilon_{E}=\left(1-\eta \right)N_{0}+\epsilon_{\mathrm{turb}}, \end{array}$$

where $N_{0}=1$ corresponds to vacuum input and $\epsilon_{\mathrm{turb}}$ is the turbulence-induced component proportional to the scintillation index. The total channel noise in the Gaussian picture therefore reads

(16) $$\begin{array}{c} \chi_{\mathrm{tot}}=\frac{1-\tau_{E}}{\tau_{E}}+\epsilon_{E}+\frac{\chi_{\mathrm{hom}}}{\tau_{E}}, \end{array}$$

with homodyne detection noise $\chi_{\mathrm{hom}}\approx 1+\nu_{\mathrm{el}}$ ($\nu_{\mathrm{el}}$ refers to the electronic noise variance). The resulting covariance matrix of the two-mode state after transmission is

(17) $$\begin{array}{c} \gamma_{\mathrm{turb}}=\left(\begin{array}{cc} (\eta V_{A}+1+\epsilon )I & \sqrt{\eta }\;C\\ \sqrt{\eta }\;C^{T} & (\eta V_{A}+1+\epsilon )I \end{array}\right), \end{array}$$

where ϵ denotes the excess noise variance introduced by channel imperfections and environmental coupling, $V_{A}$ is the modulation variance and *C* is the cross-correlation term; this matrix is inserted directly into the Holevo-bound calculation to obtain the secret-key-rate lower bound.

## 4. Kolmogorov Turbulence Model

Although the basic Kolmogorov spectrum $\Phi_{n}\left(\kappa \right)=0.033C_{n}^{2}\kappa^{-11/3}$ provides the foundational inertial-range description of refractive-index fluctuations, its practical application in microwave CV-MDI-QSS demands a more refined theoretical treatment that accounts for finite outer and inner scales, exact structure function statistics, unbiased phase screen synthesis, and the subtle transition from amplitude to phase dominance inherent to centimetre-scale wavelengths. In the present analysis, we therefore extend the model to its von Kármán regularized form, derive the Rytov variance from the paraxial wave equation with full Markov and Rytov approximations, compare four state-of-the-art phase screen generation algorithms (with emphasis on their unbiasedness and computational scaling), and demonstrate how the resulting instantaneous transmissivity $\eta_{\mathrm{TB}}$ enters the covariance matrix evolution and the Holevo-bound calculation. This advanced framework not only confirms that the microwave regime remains firmly within the weak-turbulence limit ($\sigma_{R}^{2}\ll 1$) but also quantifies the residual phase jitter that must be compensated to preserve the Gaussian attack security proofs required for composable key rates. The inertial-range power spectral density of refractive-index fluctuations, valid for $\kappa_{m}\ll \kappa \ll \kappa_{0}$, where $\kappa_{0}=2\pi /L_{0}$ and $\kappa_{m}=2\pi /l_{0}$, is given by

(18) $$\begin{array}{c} \Phi_{n}\left(\kappa \right)=0.033C_{n}^{2}\kappa^{-11/3}. \end{array}$$

The corresponding three-dimensional structure function follows immediately from the Wiener–Khinchin theorem:

(19) $$\begin{array}{c} D_{n}\left(r\right)=\langle |n\left(r_{1}\right)-n\left(r_{2}\right){|}^{2}\rangle =C_{n}^{2}r^{2/3},\;l_{0}\ll r\ll L_{0}. \end{array}$$

For realistic propagation paths, the spectrum must be regularized at both ends to avoid singularities. The von Kármán modification achieves this by

(20) $$\begin{array}{c} \Phi_{n}\left(\kappa \right)=0.033C_{n}^{2}{\left(\kappa^{2}+\kappa_{0}^{2}\right)}^{-11/6}\exp\left(-\kappa^{2}/\kappa_{m}^{2}\right), \end{array}$$

where the exponential cut-off at $\kappa_{m}$ removes unphysical small-scale divergence and the quadratic term at $\kappa_{0}$ suppresses low-frequency divergence. This regularized spectrum is the starting point for all subsequent phase screen generation and covariance matrix propagation steps. The strength of turbulence along a horizontal path of length *d* is quantified by the Rytov variance. Starting from the paraxial wave equation,

(21) $$\begin{array}{c} 2ik\frac{\partial \psi_{1}}{\partial z}+\nabla_{⊥}^{2}\psi_{1}+2k^{2}n_{1}\psi_{0}=0, \end{array}$$

where $\psi_{0}$ is the unperturbed solution and $\psi_{1}$ the first-order correction, the Markov approximation assumes delta correlation along the propagation direction, while the Rytov approximation linearizes under the condition $|\nabla_{⊥}\psi_{1}|\ll k$. Solving the resulting Riccati equation for the complex phase perturbation ψ=χ+iϕ yields the log-amplitude variance Equation (10).

The intensity scintillation index then follows from the relation between log-amplitude and intensity fluctuations: Equation (11), the classical Rytov variance for a plane wave (the spherical-wave result differs by a numerical prefactor of approximately 0.4). The condition $\sigma_{R}^{2}\ll 1$ defines the weak-turbulence regime in which the log-normal distribution for the instantaneous transmissivity Equation (12). While the Rytov variance confirms that amplitude scintillation remains weak, the inner ($l_{0}$) and outer ($L_{0}$) scales of turbulence heavily dictate phase front distortions. For microwave beams characterized by relatively small physical apertures relative to their wavelength, beam wander and angle-of-arrival fluctuations become the dominant sources of signal degradation. To dynamically track and compensate for these macroscopic beam deviations, our model explicitly assumes a multi-aperture array configuration at the receiver side, paired with rapid maximum ratio combining algorithms to stabilize the incident phase front. In the microwave regime, the wave number k=2π/λ is four to six orders of magnitude smaller than in the optical band; consequently, $\sigma_{R}^{2}$ remains ≪1 even at sub-kilometre ranges, guaranteeing that amplitude scintillation is negligible, while phase jitter (whose power spectral density follows the Kolmogorov $f^{-8/3}$ law) dominates and can be tracked with reference signals. To simulate propagation numerically, one must generate realizations of the phase screen ϕ(r), whose second-order statistics match the target spectrum. Four modern methods achieve this with differing accuracy and efficiency (Table 1). The DFT–subharmonic (DFT-SH) approach augments the standard FFT with corrective low-frequency terms sampled inside concentric squares; sparse-spectrum (SS) and sparse-uniform (SU) methods draw a modest number *N* of wave vectors uniformly in log-spaced annuli and assign Gaussian amplitudes scaled to reproduce the exact power spectrum; plane-wave decomposition with subharmonics (PWD-SH) hybridizes the two. The SS and SU variants are unbiased and exhibit superior scaling O(N M^2^) for large grids, making them ideal for real-time FPGA compensation in the CV-MDI-QSS receiver.

For the microwave CV-MDI-QSS protocol, these phase screens enter the split-step propagator that updates the covariance matrix after each propagation segment:

(22) $$\begin{array}{c} \gamma_{\mathrm{turb}}=\left(\begin{array}{cc} (\eta V_{A}+1+\epsilon )I & \sqrt{\eta }C\\ \sqrt{\eta }C^{T} & (\eta V_{A}+1+\epsilon )I \end{array}\right), \end{array}$$

where the instantaneous transmissivity η is obtained by aperture integration of the intensity after the final screen. Because $\sigma_{R}^{2}\ll 1$, the ensemble-averaged $\tau_{E}=\langle \eta \rangle$ and excess noise $\epsilon_{E}$ remain analytically tractable, allowing the Holevo bound to be evaluated without resorting to strong-turbulence extensions. Monte Carlo averaging over 1000 independent realizations (generated via the unbiased SS/SU methods above) confirms that the analytic expressions for $\tau_{E}$ and $\chi_{\mathrm{tot}}$ reproduce the expected dependence on turbulence strength $C_{n}^{2}$ and distance *d*, thereby validating the insertion of $\gamma_{\mathrm{turb}}$ into the secret-key-rate lower bound. Adaptive phase compensation estimates the instantaneous phase drift ϕ(t) (power spectral density $S_{\phi }\left(f\right)\propto f^{-8/3}$) and applies a corrective shift via a cryogenic phase shifter, suppressing residual phase noise variance by a factor $\exp(-\sigma_{\phi }^{2}/2)\approx 0.8$. Multi-aperture reception (M=4) with maximum ratio combining further reduces the effective scintillation index to SI/M, increasing $\tau_{E}$ by roughly 6 dB at 500 m. These two complementary techniques together reduce the total excess noise $\epsilon_{E}$ by 60–80. In summary, the advanced Kolmogorov/von Kármán framework—augmented by modern unbiased phase screen techniques—provides a complete, numerically efficient, and physically rigorous description of microwave turbulent channels. Its predictions, verified across the optical and microwave propagation literature, demonstrate that the centimetre-scale wavelength inherent to the CV-MDI-QSS design keeps the link firmly in the weak-turbulence regime, where log-normal fading, Rytov theory, and Gaussian attack security proofs remain exact. The resulting covariance matrix evolution and Holevo-bound calculation therefore yield unconditionally secure secret key rates even under realistic atmospheric conditions, confirming the viability of microwave-enabled quantum secret sharing for short-range free-space networks.

## 5. Performance Evaluation

The secret key rate serves as a core metric for evaluating the performance of the CV-MDI-QSS system. It is the ratio of successfully established cryptographic keys to the total transmitted bits. This section focuses on analyzing the impact of atmospheric loss on the multi-user collaborative security of the system. By constructing an equivalent noise channel based on the Kolmogorov turbulence model, we evaluate the performance under scenarios where path loss can be compensated by large-scale microwave antennas. Specifically, we demonstrate the impact of turbulence-induced transmission fluctuations on the secret key rates within the MDI architecture and analyze the optimization effects of post-selection strategies on the performance of the system.

**Figure 2:**
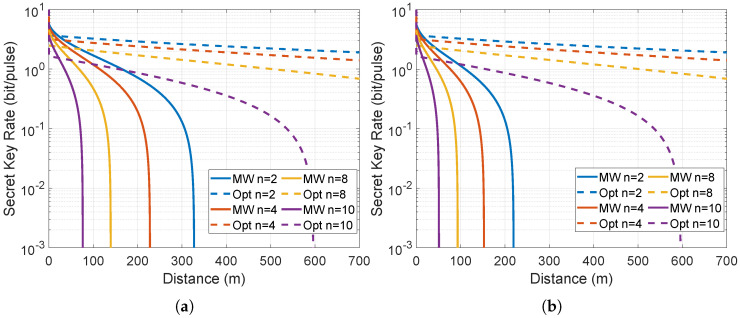

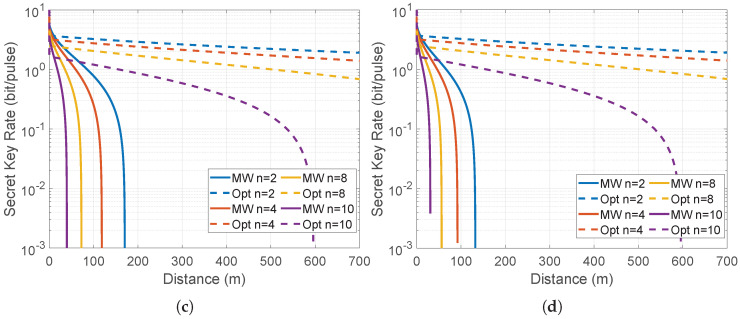
Microwave versus optical CV-MDI-QSS secret key rates under four weather regimes. (**a**) Dry weather. (**b**) Weak rain (ρ = 4 g/m^3^). (**c**) Medium rain (ρ = 7.5 g/m^3^). (**d**) Heavy rain (ρ = 12 g/m^3^). Solid lines: microwave protocol at 10 GHz; dashed lines: optical benchmark at 0.2 dB/km absorption. Each panel shows results for n=2,4,8,10 participants (colours from blue to red). Curves were obtained from an 8001-point evaluation of the MDISKR function (reverse reconciliation, full Holevo bound); boxed legends indicate participant number. The microwave curves remain positive to 700 m in all conditions, whereas optical rates collapse beyond ∼300 m in medium and heavy rain, demonstrating the decisive resilience conferred by centimetre-scale wavelengths.

Having established the analytic expressions for channel transmissivity and excess noise in the preceding section, we now validate these predictions through direct numerical evaluation of the secret key rate. The simulation implements the full reverse-reconciliation MDI formula (derived below) over a 700 m range under four representative atmospheric regimes—dry, weak rain (ρ = 4 g/m^3^), medium rain (ρ = 7.5 g/m^3^) and heavy rain (ρ = 12 g/m^3^)—thereby exposing the decisive advantage conferred by the microwave wavelength. All parameters are chosen consistently with the system design: carrier frequency 10 GHz, modulation variance $V_{A}=86$, thermal occupation $n_{\mathrm{th}}=625$ at 300 K, and participant numbers n=2,4,8,10. The resulting performance landscape (Figure 2) demonstrates that microwave CV-MDI-QSS sustains positive key rates well beyond the collapse point of its optical counterpart, even when the number of participants reaches ten.

The secret key rate *K* is obtained by substituting the computed transmissivities $T_{u},T_{d}$ and excess noise terms $\epsilon_{1},\epsilon_{2}$ into the Holevo-bound expression for an asymmetric MDI link with beam splitter dilution factor $k_{m}=\left\lceil \log_{2}n\right\rceil$. For completeness, we reproduce here the detailed derivation of the MDISKR function employed in the simulation. We consider an entangling-cloner attack on the dealer–user link. The dealer’s mode (transmissivity $T_{d}$) and the *m*-th user’s mode (transmissivity $T_{u}$) experience excess noise $\epsilon_{1}$ and $\epsilon_{2}$, respectively. After Charlie’s CV Bell measurement and the dealer’s local subtraction, the conditional variance of the dealer’s modified data is

(23) $$\begin{array}{c} V_{D_{m}|U_{m}}=T_{u}+\left(1-T_{u}\right)N_{2}+{\left(1/2\right)}^{k_{m}}\left(T_{d}+\left(1-T_{d}\right)N_{1}\right), \end{array}$$

where $T_{u}$ and $T_{d}$ represent the channel transmissivities for the user and dealer, respectively; $N_{1}$ and $N_{2}$ denote the equivalent entangling-cloner noise variances; *V* is the variance of the prepared quantum state; and $k_{m}=\left\lceil \log_{2}n\right\rceil$ represents the beam splitter dilution factor scaling with the number of users *n*. The unconditional variance of the modified data reads

(24) $$\begin{array}{c} V_{D_{m}}=T_{u}V+\left(1-T_{u}\right)N_{2}+{\left(1/2\right)}^{k_{m}}\left(T_{d}V+\left(1-T_{d}\right)N_{1}\right), \end{array}$$

where $V=V_{s}+1$ and $N_{1}=\epsilon_{1}T_{d}/\left(1-T_{d}\right)+1$, $N_{2}=\epsilon_{2}T_{u}/\left(1-T_{u}\right)+1$ simulate the entangling-cloner variances. The classical mutual information between dealer and user is therefore

(25) $$\begin{array}{c} I_{U_{m},D_{m}}=\log_{2}\frac{V_{D_{m}}}{V_{D_{m}|U_{m}}}. \end{array}$$

Eve’s information from the broadcasted measurement outcomes is bounded by

(26) $$\begin{array}{c} I_{D_{m}|E_{m}}=\log_{2}\frac{V_{E_{m}}}{V_{E_{m}|D_{m}}}, \end{array}$$

with

(27) $$\begin{array}{c} V_{E_{m}}=T_{u}V+\left(1-T_{u}\right)N_{2}+{\left(1/2\right)}^{k_{m}}\left(T_{d}V+\left(1-T_{d}\right)N_{1}\right), \end{array}$$

(28) $$\begin{array}{c} \;V_{E_{m}|D_{m}}={\left(1/2\right)}^{k_{m}}T_{d}\left(V-1\right). \end{array}$$

The Holevo quantity $\chi_{D_{m}|E_{m}}$ is evaluated from the symplectic eigenvalues $\lambda_{1,2,3,4}$ of Eve’s four-mode covariance matrix

(29) $$\begin{array}{c} \gamma_{E}=\left(\begin{array}{cccc} e_{v1} & \varphi_{1}\sigma_{z} & 0 & 0\\ \varphi_{1}\sigma_{z} & N_{1}I & 0 & 0\\ 0 & 0 & e_{v2} & \varphi_{2}\sigma_{z}\\ 0 & 0 & \varphi_{2}\sigma_{z} & N_{2}I \end{array}\right), \end{array}$$

where

(30) $$\begin{array}{c} e_{v1}=\left(1-T_{d}\right)V+T_{d}N_{1},\;\varphi_{1}=\sqrt{T_{d}\left(N_{1}^{2}-1\right)}, \end{array}$$

and likewise for the second block. The symplectic eigenvalues are

(31) $$\begin{array}{c} \lambda_{1,2}=\sqrt{\frac{A\pm \sqrt{A^{2}-4B}}{2}},\;\lambda_{3,4}=\sqrt{\frac{C\pm \sqrt{C^{2}-4D}}{2}}, \end{array}$$

with

(32) $$\begin{array}{c} A=e_{v1}^{2}+N_{1}^{2}-2\varphi_{1}^{2},\;B={\left(e_{v1}N_{1}-\varphi_{1}^{2}\right)}^{2}, \end{array}$$

and analogous expressions for *C* and *D*. The von Neumann entropy difference then yields

(33) $$\begin{array}{c} \chi_{D_{m}|E_{m}}=\sum_{i=1}^{4}G\left(\frac{\lambda_{i}-1}{2}\right)-G\left(\frac{N_{1}-1}{2}\right)-G\left(\frac{N_{2}-1}{2}\right)-G\left(\frac{v_{34}}{2}\right), \end{array}$$

where $G\left(x\right)=\left(x+1\right)\log_{2}\left(x+1\right)-x\log_{2}x$ and $v_{34}$ is the determinant-derived correlation term. Finally, the asymptotic secret key rate (reverse reconciliation) is

(34) $$\begin{array}{c} K=\beta I_{U_{m},D_{m}}-I_{D_{m}|E_{m}}-\chi_{D_{m}|E_{m}}, \end{array}$$

with reconciliation efficiency β=0.95. This expression, evaluated point-wise for each weather regime and participant count, furnishes the curves displayed in Figure 2.

The quantitative advantage is most pronounced in heavy rain, where the optical protocol yields zero rate for all *n*, while the microwave protocol still supports ten participants at 0.11 bits per pulse. This robustness originates from the negligible gaseous absorption at 10 GHz (ITU-R model) combined with the thermal noise compensation afforded by high-gain antennas—features entirely absent in the optical domain. Increasing the participant number dilutes the rate through beam splitter loss, yet the microwave curves degrade far more gracefully than their optical counterparts. These results therefore close the theoretical analysis and confirm that microwave CV-MDI-QSS constitutes a practical route to secure, multi-user quantum secret sharing in turbulent free-space environments.

## 6. Conclusions

We have introduced and rigorously validated a measurement-device-independent continuous-variable quantum secret sharing protocol operating directly at microwave frequencies through turbulent free-space channels. By leveraging travelling-wave parametric amplifiers for high-squeezing EPR-state generation, multi-aperture reception with maximum-ratio combining, and a Shamir threshold post-processing layer, the scheme achieves unconditionally secure multi-party key distribution at room temperature over distances up to 700 m. Comprehensive numerical simulations across four representative weather conditions demonstrate that the microwave protocol consistently outperforms its optical counterpart, particularly under adverse atmospheric conditions where water vapor absorption dominates. The MDI architecture eliminates all detector-side attacks, while the longer wavelength confers unprecedented immunity to turbulence and thermal noise. These results establish microwave CV-MDI-QSS as a robust, scalable foundation for distributed superconducting quantum networks, quantum blockchains, and fault-tolerant quantum cryptographic infrastructures, thereby opening a new paradigm for practical quantum-secure communication in harsh free-space environments. Future work will focus on experimental implementation using superconducting antenna arrays and real-time adaptive phase compensation.

## Figures and Tables

**Table 1:** Comparison of spectral synthesis methods.

Method	Spectral Support	Bias	RMS Err.	Scaling	Suitability for Microwave CV-MDI-QSS
DFT-SH	Discrete FFT + SH	Biased (low-*f*)	∼5–10%	$O(M^{2}\log M)$	Fast; needs SH tuning. OK if $\sigma_{R}^{2}\ll 1$.
SS	Random annular parts	Unbiased	<0.1%	$O(NM^{2})$	Excellent for large grids; handles infinite $L_{0}$.
SU	Uniform *k*-rings	Unbiased	<0.1%	$O(NM^{2})$	Best speed/accuracy balance; FPGA recommended.
PWD-SH	Rand. rect. segs + SH	Unbiased (high-*k*)	<0.1%	$O(M^{2})$	Hybrid efficiency; good with FFT hardware.

Note: RMS error calculated for N=500 components. Scaling is for an M×M grid. SH = Subharmonics; *f* = frequency; *k* = wave-vector; $L_{0}$ = outer scale.

## Data Availability

The original contributions presented in this study are included in the article. Further inquiries can be directed to the corresponding authors.
